# Isolation and Functional Characterization of Calcitonin-Like Diuretic Hormone Receptors in *Rhodnius prolixus*

**DOI:** 10.1371/journal.pone.0082466

**Published:** 2013-11-29

**Authors:** Meet Zandawala, Shizhong Li, Frank Hauser, Cornelis J. P. Grimmelikhuijzen, Ian Orchard

**Affiliations:** 1 Department of Biology, University of Toronto Mississauga, Mississauga, Ontario, Canada; 2 Center for Functional and Comparative Insect Genomics, Department of Biology, University of Copenhagen, Copenhagen, Denmark; University of Rouen, France, France

## Abstract

Several families of diuretic hormones exist in insects, one of which is the calcitonin-like diuretic hormone (CT/DH) family. CT/DH mediates its effects by binding to family B G-protein coupled receptors (GPCRs). Here we isolate and functionally characterize two *R. prolixus* CT/DH receptor paralogs (Rhopr-CT/DH-R1 and Rhopr-CT/DH-R2) using a novel heterologous assay utilizing a modified human embryonic kidney 293 (HEK293) cell line. Rhopr-CT/DH-R1 is orthologous to the previously characterized *D. melanogaster* CT/DH receptor (CG17415) while Rhopr-CT/DH-R2 is orthologous to the *D. melanogaster* receptor (CG4395), an orphan receptor whose ligand was unknown until now. We determine the cDNA sequences of three splice variants encoding Rhopr-CT/DH-R1 (*Rhopr-CT/DH-R1-A*, *Rhopr-CT/DH-R1-B* and *Rhopr-CT/DH-R1-C*) and two splice variants encoding Rhopr-CT/DH-R2 (*Rhopr-CT/DH-R2-A* and *Rhopr-CT/DH-R2-B*). *Rhopr-CT/DH-R1-A* and *Rhopr-CT/DH-R2-A* encode truncated receptors that lack six and seven of the characteristic seven transmembrane domains, respectively. Rhopr-CT/DH-R1-B and Rhopr-CT/DH-R1-C, which only differ by 2 amino acids in their C-terminal domain, can both be activated by Rhopr-CT/DH at equal sensitivities (EC_50_ = 200-300nM). Interestingly, Rhopr-CT/DH-R2-B is much more sensitive to Rhopr-CT/DH (EC_50_ = 15nM) compared to Rhopr-CT/DH-R1-B/C and also yields a much greater response (amplitude) in our heterologous assay. This is the first study to reveal that insects possess at least two CT/DH receptors, which may be functionally different. Quantitative PCR demonstrates that *Rhopr-CT/DH-R1* and *Rhopr-CT/DH-R2* have distinct expression patterns, with both receptors expressed centrally and peripherally. Moreover, the expression analysis also identified novel target tissues for this neuropeptide, including testes, ovaries and prothoracic glands, suggesting a possible role for Rhopr-CT/DH in reproductive physiology and development.

## Introduction

Various neurohormone families have been implicated in regulating diuresis in insects. One such family is the calcitonin-like diuretic hormone (CT/DH) family which is related to the mammalian calcitonin and calcitonin gene-related peptide hormonal system. The first member of this peptide family in insects was isolated and functionally characterized in *Diploptera punctata* [1]. This peptide was originally termed diuretic hormone 31 (DH_31_) due to its ability to stimulate Malpighian tubule (MT) secretion in certain insects and due to the fact that it is comprised of 31 amino acids [1-3]. As is the case with many peptides that are named because of a particular bioassay involved in their isolation, regulating diuresis may not be their function in other insects. Thus, CT/DHs do not stimulate MT secretion in *Acrosternum hilare* and *Podisus maculiventris* [4]. Moreover, the role of *Rhodnius prolixus* CT/DH (Rhopr-CT/DH) in diuresis is also questionable as it does not stimulate water reabsorption across the midgut, and only stimulates MT secretion at a rate which is 1.5% of maximum [5,6]; however, it may play a broad role in feeding-related physiological events in various insects. For example, Rhopr-CT/DH has been shown to have myostimulatory effects on hindgut, dorsal vessel and salivary glands whereas the *Drosophila melanogaster* CT/DH is required for peristalsis in the larval midgut [7-9]. Furthermore, *D. punctata* CT/DH analogs have anorexigenic effects in *Locusta migratoria* nymphs [10]. It is thus evident that CT/DHs, like several other neuropeptides, are pleiotropic in nature. Hence, in order to elucidate additional physiological roles for these hormones, it is important to identify and characterize their receptors and determine their expression patterns. 

Insect CT/DH receptors (CT/DH-Rs) belong to the family of secretin-like (family B 1) G-protein coupled receptors (GPCRs) [11]. Johnson et al. characterized the first insect CT/DH-R from *D. melanogaster* in 2005 [12]. Signaling through this receptor was shown to be dependent on accessory proteins (receptor activity modifying proteins (RAMPs) and receptor component protein (RCP)), in a manner analogous to mammals [13,14]. Recently, a receptor orthologous to this was functionally characterized in *Aedes aegypti* (*Aedae*GPCRCAL1) via RNAi-based knockdown [15,16]. RNAi treated females showed a 30% reduction in fluid excretion (relative to control groups) following a blood meal and the hindguts exhibited a 50% reduction in contraction frequency in response to *A. aegypti* CT/DH (Aedae-CT/DH) compared to controls. Moreover, a 57% decrease in fluid secretion in response to Aedae-CT/DH was also observed in MTs in which *Aedae*GPCRCAL1 was knocked-down [15]. 

In the present study, we have isolated and characterized a CT/DH-R from *R. prolixus* that is orthologous to the previously characterized CT/DH-Rs in *D. melanogaster* and *A. aegypti* [12,15]. We propose to rename these receptors as CT/DH-R1. Moreover we have also isolated and characterized another family B1 GPCR from *R. prolixus* that is orthologous to the *D. melanogaster* receptor (CG4395), *hector*. This orphan receptor is also related to insect CT/DH-Rs and is activated by Rhopr-CT/DH. Hence, we propose to name these receptors as CT/DH-R2. Rhopr-CT/DH-R2 is much more sensitive to Rhopr-CT/DH compared to Rhopr-CT/DH-R1 in our heterologous assay utilizing human embryonic kidney (HEK)-293 cells stably expressing a modified cyclic nucleotide-gated (CNG) channel (HEK293/CNG). We obtained robust and sensitive responses in these cells without having to co-express any accessory proteins, making it ideal to study CT/DH-Rs and, perhaps, deorphanize other family B1 GPCRs. To our knowledge, this is the first study to reveal that insects possess at least two CT/DH receptors, which may be functionally different. Quantitative PCR demonstrates that *Rhopr-CT/DH-R1* and *Rhopr-CT/DH-R2* have distinct expression patterns, with both receptors expressed centrally and peripherally. 

## Materials and Methods

### Animals

Fifth-instar and adult *R. prolixus* (4-5 weeks post-feeding) were raised in a long standing colony that was maintained in incubators at 60% humidity and 25°C. The insects were routinely fed artificially once in each instar on defibrinated rabbit blood (Hemostat Laboratories, Dixon, CA, USA; supplied by Cedarlane Laboratories Inc., Burlington, ON, Canada). 

### Isolation of cDNA sequences encoding R. prolixus **CT**/**DH** receptors

Supercontigs in FASTA format, representing the *R. prolixus* preliminary genome assembly (June 2009 release), were downloaded from the genome server at The Genome Institute at Washington University (http://genome.wustl.edu/pub/organism/Invertebrates/Rhodnius_prolixus/). These supercontigs were then imported into Geneious Pro 4.7.6 and used to perform local tBLASTn search, with the *D. melanogaster* CT/DH receptor (CG17415, accession no: NP_725278.1) protein sequence acting as the query. Hits along two different supercontigs were obtained; these represent two putative CT/DH receptors. Primers specific to the hit regions were designed (Table S1 in File S1) and used to amplify the partial cDNA sequence encoding Rhopr-CT/DH-R1 and Rhopr-CT/DH-R2. Template for the PCR was cDNA synthesized using total RNA extracted from individually-dissected tissues (see section: Quantitative PCR tissue profiling). PCR was performed using s1000 thermal cycler (Bio-Rad Laboratories, Mississauga, ON, Canada) with a temperature-cycling profile that consisted of an initial denaturation (94°C for 3 min) and 35 cycles of denaturation (94°C for 30 sec), annealing (59°C for 30 sec) and extension (72°C for 1 min); a final 10 min extension at 72°C was also included. Gel electrophoresis was used to visualize the PCR product which was then extracted using the EZ-10 Spin Column DNA Gel Extraction Kit (Bio Basic Inc., Markham, ON, Canada). The gel extracted product was cloned and sequenced using the methods described earlier [17]. 

Complete cDNA sequences encoding the two receptors were obtained using a modified 5´ and 3´ rapid amplification of cDNA ends (RACE) PCR technique, as described earlier [17]. Primers used for 5´ and 3´ RACE PCRs have been listed in Table S2 and Table S3 in File S1, respectively. Lastly, the largest cDNA fragments encoding the receptors were amplified using the primers listed in Table S4 in File S1 and a proof-reading Taq polymerase. The PCR products were cloned and sequenced as explained earlier [17]. 

### Sequence analysis

The intron-exon boundaries were predicted using a combination of a BLAST search of the *R. prolixus* genome and Genie, an online software for splice site prediction [18]. Membrane topology of the receptors was predicted using the Transmembrane Prediction Tool plugin for Geneious. The potential phosphorylation sites were predicted using the NetPhos 2.0 Server [19] and the potential N-linked glycosylation sites predicted using the NetNGlyc 1.0 Server. Clustal Omega (http://www.ebi.ac.uk/Tools/msa/clustalo/ - last accessed on August 1, 2013) was used to align Rhopr-CT/DH-R1 (KC660148, KC660149 and KC660150) and R2 isoforms (KF446640 and KF494337) with its homologs from *D. melanogaster* (NP_725278.1 and NP_572843.2) and *Aedes aegypti* (AEU12191.1). The alignment figure was obtained using the BOXSHADE 3.21 server (http://www.ch.embnet.org/software/BOX_form.html - last accessed on August 1, 2013).

Additional family B1 GPCR amino acid sequences were included for phylogenetic analysis. These included corticotropin releasing-factor (CRF)-related diuretic hormone (CRF/DH) receptors (CRF/DH-Rs), pigment dispersing factor (PDF) receptors (PDF-Rs) and CT/DH-Rs from a variety of insects. Moreover, CRF receptors (NP_001138618.1 and NP_001189404.1), calcitonin receptor (CTR) (NP_001158209.1) and calcitonin receptor-like receptor (CRLR) (NP_001258680.1) from *Homo sapiens* were also included in the analysis and *D. melanogaster* metabotropic glutamate receptor (NP_524639.2) was utilized as an outgroup. ClustalX2 was used to align these sequences and the alignment exported to MEGA5 [20,21]. A maximum parsimonious tree was constructed using Close-Neighbor-Interchange (CNI) analysis and the bootstrap values obtained were based on 1000 replicates.

### Preparation of expression vectors

The largest cDNA fragments encoding *Rhopr-CT/DH-R1* transcript variants and *Rhopr-CT/DH-R2-B* were amplified as described earlier (see section: Isolation of cDNA sequences encoding *R. prolixus* CT/DH receptors). The PCR products from these reactions were used as a template to amplify the ORF and introduce a Kozak translation initiation sequence at the 5´ end using the primers listed in Table S5 in File S1. The resulting products were cloned into pGEM-T Easy vector (Promega, Madison, WI, USA). These were then subcloned into either pIRES2-ZsGreen1 (Clontech, Mountain View, CA, USA) or pcDNA 3.1^+^ (Life Technologies Corporation, Carlsbad, CA, USA) for expression in mammalian cells. 

### Cell culture and transfections

Human embryonic kidney (HEK)-293 cells stably expressing a modified cyclic nucleotide-gated (CNG) channel (HEK293/CNG) (previously available through BD Biosciences, Mississauga, ON, Canada) were used to functionally characterize the receptors [22]. HEK293/CNG cells were grown in Dulbecco’s Modified Eagle Medium Nutrient Mixture F12-Ham (DMEM/F12) and supplemented with 10% heat-inactivated fetal bovine serum (FBS), 1% penicillin and streptomycin and 250µg/mL G418 (Life Technologies Corporation, Carlsbad, CA, USA). The cells were incubated at 37°C in 5% CO_2_. X-tremeGENE HP DNA transfection reagent (Roche Applied Science, Indianapolis, IN, USA) was used to transiently co-transfect the cells with the expression vectors containing receptor transcript variant and cytoplasmic luminescent reporter aequorin at ratio of 2:1 (transfection reagent to expression vectors) using the manufacturer recommended protocol. For negative control, empty expression vector without any receptor transcript was also used to transfect the cells. Cells were incubated for 48 hours and then used to perform the bioluminescence assay. 

Alternatively spliced transcript variants of mammalian calcitonin receptors have been known to dimerize with normal functional receptors and inhibit either their surface expression or ligand-induced intracellular cAMP production [23,24]. To determine if Rhopr-CT/DH-R1-A interacted with Rhopr-CT/DH-R1-B/C, we utilized Chinese hamster ovary (CHO) cells stably expressing the human G-protein G16 (CHO/G16) [25]. CHO/G16 cells stably expressing either Rhopr-CT/DH-R1-B or Rhopr-CT/DH-R1-C were grown and transfected as described earlier [26]. These cells were then transiently co-transfected with Rhopr-CT/DH-R1-A and aequorin [26].

### Bioluminescence assay

Bioluminescence assay using CHO/G16 cells was performed as described previously [25-27]. To perform the assay using HEK293/CNG cells, they were first harvested by incubating in a PBS-EDTA solution and resuspended in bovine serum albumin (BSA) media (DMEM/F12 containing 1% BSA and 1% penicillin and streptomycin). Coelenterazine h (Promega, Madison, WI, USA) was then added to the cells at a 5µM final concentration and incubated for 3 hours with stirring in the dark. The cells were then diluted 10-fold using BSA media and used to perform the assay. Various doses of peptides were prepared in BSA media and plated in triplicates across a 96-well plate. Cells were loaded in each well using an automated injector and the luminescence recorded over 20 seconds using a Wallac Victor2 plate reader (Perkin Elmer, San Diego, CA, USA). Rhopr-CT/DH (GLDLGLSRGFSGSQAAKHLMGLAAANYAGGPamide) and Rhopr-CRF/DH (MQRPQGPSLSVANPIEVLRSRLLLEIARRRMKEQDASRVSKNRQYLQQIamide) used for the assay was custom synthesised by GenScript (Piscataway, NJ, USA) at > 95% purity. *D. melanogaster* PDF (NSELINSLLSLPKNMNDAamide) was custom synthesized by GeneMed Synthesis (San Antonio, TX, USA) at > 95% purity. Dose-response curves were obtained and the EC_50_ values determined using Prism5 software. 

### Quantitative PCR tissue profiling

The following tissues were individually-dissected from fifth-instar *R. prolixus* of both sexes and used for spatial expression analysis: (1) CNS, (2) dorsal vessel, (3) fat body, abdominal nerves, diaphragms and trachea, (4) foregut, (5) salivary glands, (6) anterior midgut, (7) posterior midgut, (8) MTs, (9) hindgut, (10) immature testes, (11) immature ovaries and (12) prothoracic glands. The following tissues were dissected from adult *R. prolixus* to determine the expression pattern in reproductive tissues: (1) testes (2) rest of the male reproductive tissues (3) ovaries and (4) rest of the female reproductive tissues. Total RNA was isolated from these tissues using PureLink® RNA Mini Kit (Life Technologies Corporation, Carlsbad, CA, USA) which was then used to synthesize cDNA using iScript™ Reverse Transcription Supermix for RT-qPCR (Bio-Rad Laboratories Ltd., Mississauga, ON, Canada). This cDNA was diluted 10-fold and subsequently used as a template for the qPCR reaction. Primers specific for each receptor variant were designed over exon-exon boundaries to determine expression levels for each transcript (Table S6 in File S1). Since the difference between *Rhopr-CT/DH-R1-B* and *Rhopr-CT/DH-R1-C* cDNA sequences was minor (see section: Rhopr-CT/DH receptors), primers differentiating these two transcripts could not be designed. The primer efficiencies for each target were calculated and delta-delta Ct method was used to determine the relative expression of each transcript. Geometric averaging of the transcript levels of three housekeeping genes (alpha-tubulin, beta-acting and ribosomal protein 49) was used to normalize the expression levels of the receptor transcripts. Experiments were performed using MX4000 Quantitative PCR System (Stratagene, Mississauga, ON, Canada) with a temperature-cycling profile that consisted of an initial denaturation (95°C for 30 sec) and 40 cycles of denaturation (95°C for 5 sec) and annealing/extension (60°C for 24 sec); this was followed by a melt curve analysis (60°C - 95°C). SsoFastTM EvaGreen® Supermix with Low ROX (Bio-Rad Laboratories Ltd., Mississauga, ON, Canada) was used to perform all experiments, which included a no template control and 2 technical replicates per reaction. Reactions for each target were run on a gel to confirm the amplicon size. The products were also verified by sequencing. 

## Results

### Rhopr-CT/DH receptors

**Figure 1:**
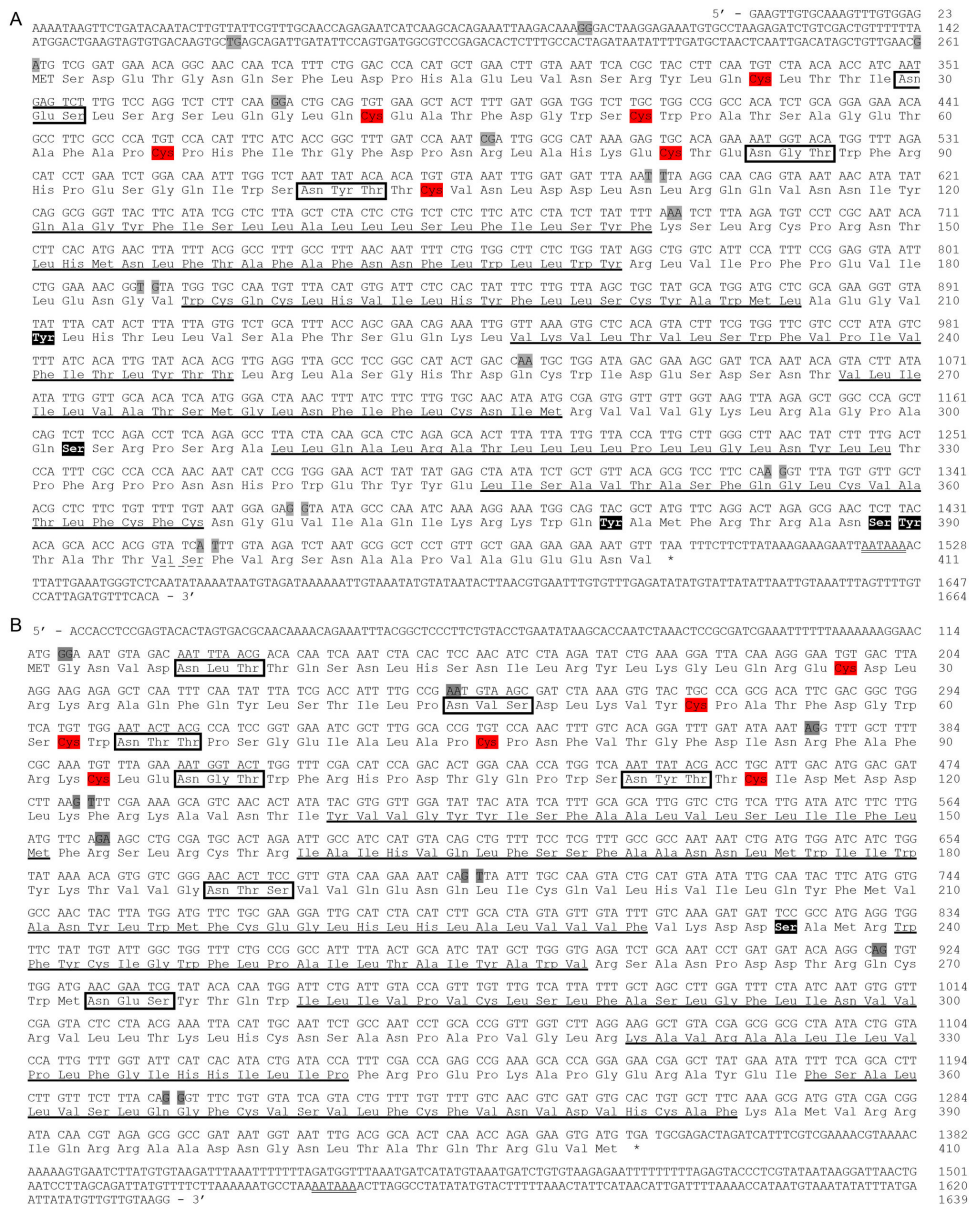
cDNA sequences and the deduced amino acid sequences of Rhopr-CT/DH-Rs. *Rhopr-CT/DH-R1-B* (A) and *Rhopr-CT/DH-R2-B* (B). The numbering for each sequence is shown at right. Within the nucleotide sequence, the exon-exon boundaries are shaded in gray and the potential polyadenylation signal is double-underlined. Within the amino acid sequence, the initial methionine start codon has been capitalized, the six conserved cysteine residues are shaded in red, the potential phosphorylation sites are shaded in black, the potential N-linked glycosylation sites are boxed and the seven predicted transmembrane domains are underlined. The two amino acid residues (valine and serine) that are absent in Rhopr-CT/DH-R1-C are dash underlined in Rhopr-CT/DH-R1-B.

**Figure 2:**
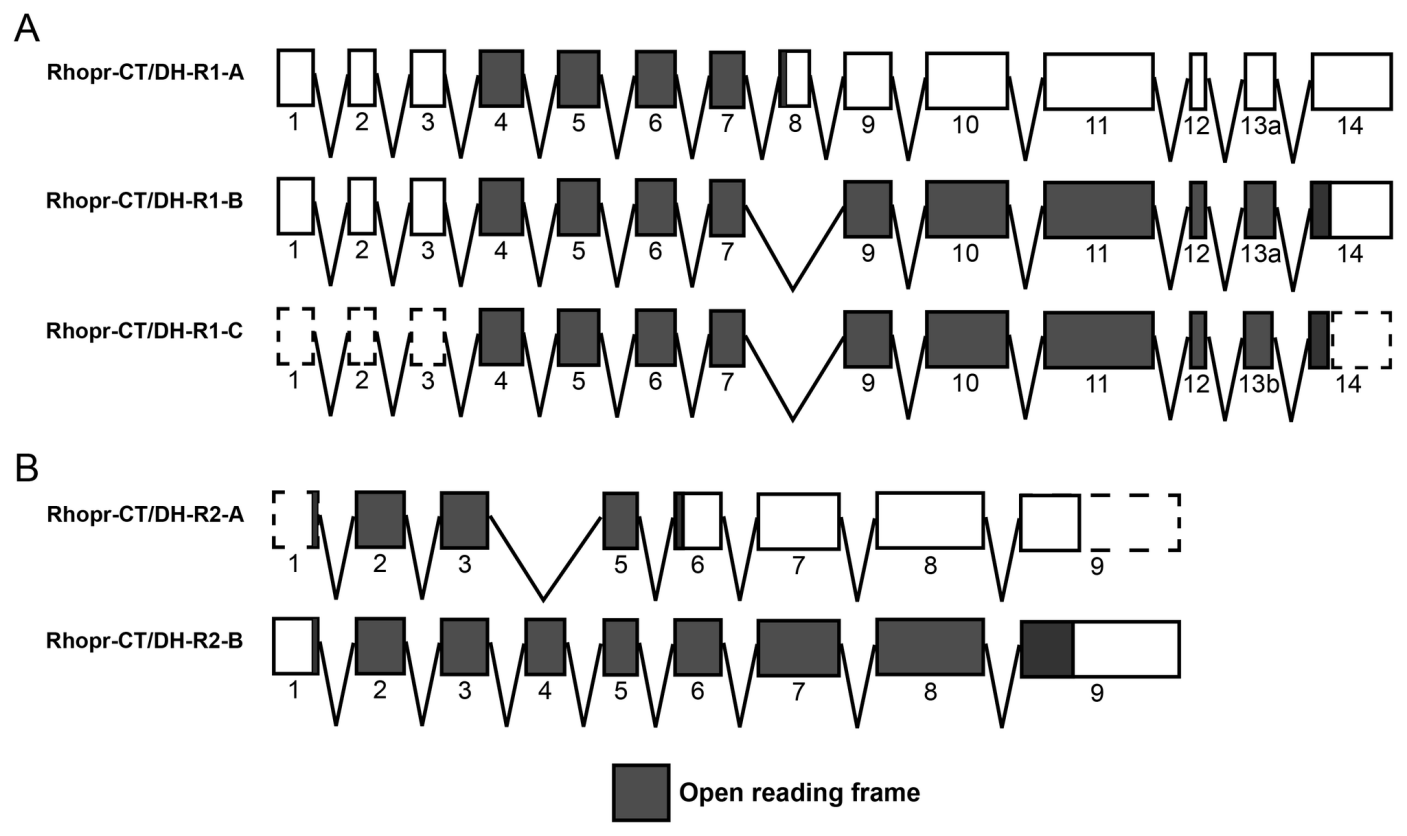
*Rhopr-CT/DH-R1* and *Rhopr-CT/DH-R2* splicing. Molecular organization of *Rhopr-CT/DH-R1* (A) and *Rhopr-CT/DH-R2* (B) splice variants based on BLAST analysis and splice site prediction. The boxes represent exons (drawn to scale). The regions shaded in gray represent the open reading frame while the unshaded regions represent the untranslated regions. The dashed boxes represent predicted regions that were not cloned.

We have isolated cDNA sequences for three splice variants encoding Rhopr-CT/DH-R1 (*Rhopr-CT/DH-R1-A*, *Rhopr-CT/DH-R1-B* and *Rhopr-CT/DH-R1-C*) and two splice variants encoding Rhopr-CT/DH-R2 (*Rhopr-CT/DH-R2-A* and *Rhopr-CT/DH-R2-B*). *Rhopr-CT/DH-R1-A*, *Rhopr-CT/DH-R1-B* and *Rhopr-CT/DH-R1-C* are 1746, 1664 and 1301 nucleotides long and encode receptors comprising of 143, 411 and 409 amino acids, respectively (Figure S1 in File S1 and Figure 1A). The untranslated regions for *Rhopr-CT/DH-R1-C* could not be cloned via RACE PCRs as primers differentiating between *Rhopr-CT/DH-R1-B* and *Rhopr-CT/DH-R1-C* could not be designed. All three variants contain a polyadenylation signal sequence in their 3´ UTR. Rhopr-CT/DH-R1-B, Rhopr-CT/DH-R1-C and Rhopr-CT/DH-R2-B all contain seven transmembrane domains, an extracellular N-terminus and an intracellular C-terminus, typical of all GPCRs (Figure 1A, B). They also contain 6 highly-conserved cysteine residues (typical of family B1 GPCRs) and 3 predicted N-linked glycosylation sites in their N-terminus, and various predicted phosphorylation sites in their intracellular domains. Rhopr-CT/DH-R1-A is a truncated version of the receptor and only contains the extracellular N-terminus and a single transmembrane domain (Figure S1 in File S1). The gene encoding this truncated receptor comprises of 14 exons that are separated by 13 introns (Figure 2A). *Rhopr-CT/DH-R1-A* contains exon 8 that is absent in the other two variants and results in a premature stop codon. The ORF for this variant spans across exons 4 to 8. *Rhopr-CT/DH-R1-B* and *Rhopr-CT/DH-R1-C* differ by only 6 nucleotides within their ORF, which results in a 2 amino acid difference between these variants within their intracellular C-terminal domain. *Rhopr-CT/DH-R1-C* utilizes an alternate splice site in exon 13 which results in that exon being 6 nucleotides shorter at the 3´ end. The ORF for these two variants spans across exons 4 to 14. 

*Rhopr-CT/DH-R2-A* and *Rhopr-CT/DH-R2-B* are 1146 and 1639 nucleotides long and yield proteins comprising of 122 and 410 amino acids, respectively (Figure S2 in File S1 and Figure 1B). Rhopr-CT/DH-R2-A comprises a partial N-terminus (contains only 5 cysteine residues) and lacks the seven transmembrane domains (Figure S2 in File S1). Rhopr-CT/DH-R2-B, on the other hand, contains all the characteristics of a family B1 GPCR (Figure 1B). Moreover, it contains 7 predicted N-linked glycosylation sites and one predicted phosphorylation site. The gene encoding these 2 receptor variants is made up of 9 exons (Figure 2B). Exon 4 is absent in *Rhopr-CT/DH-R2-A* which results in a frame shift and truncated ORF. The ORF for this variant spans across exons 1 to 6 whereas the one for *Rhopr-CT/DH-R2-B* spans across all 9 exons. The untranslated regions for *Rhopr-CT/DH-R2-A* could not be cloned due to its low expression. 

### Functional receptor assay

**Figure 3:**
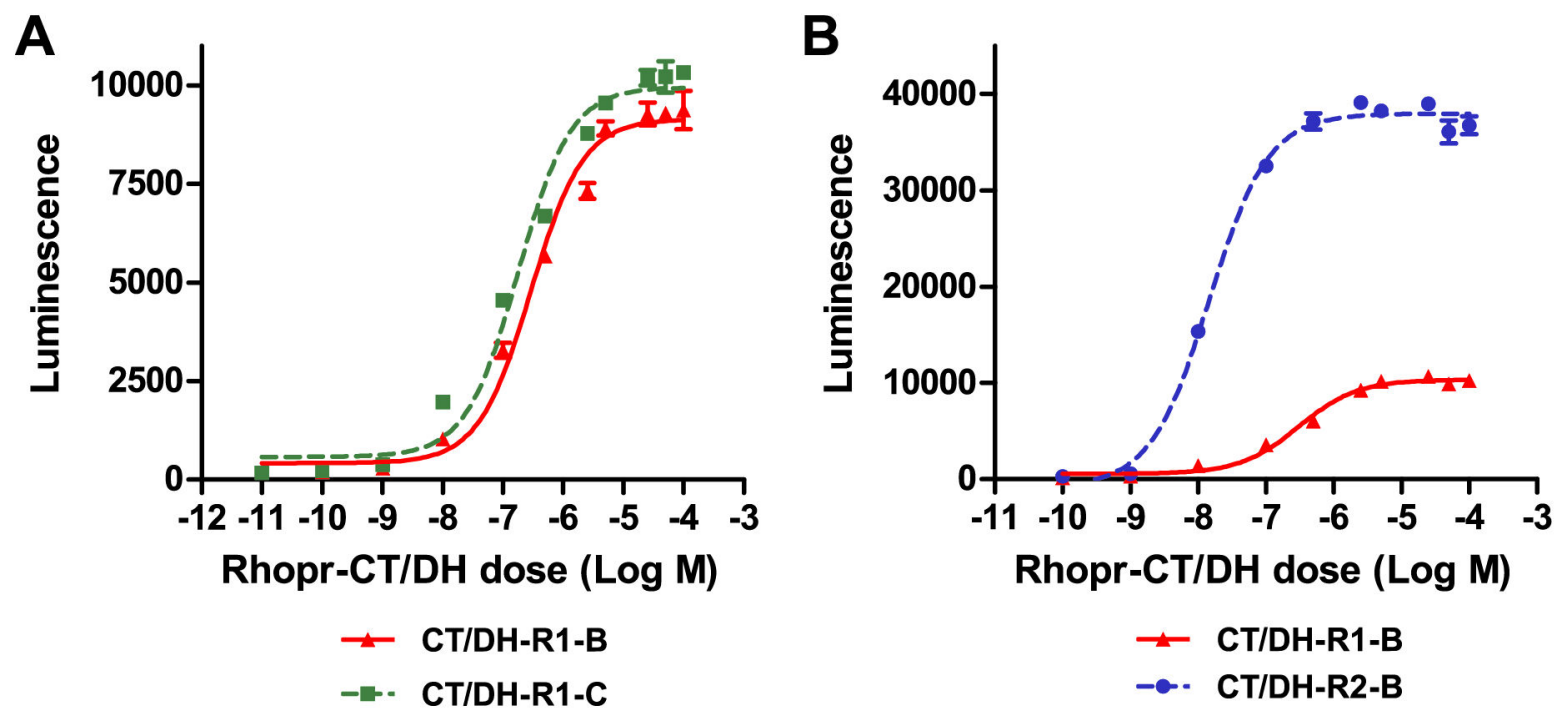
Functional assay of *R*. ***prolixus*CT/DH receptor isoforms (Rhopr-CT/DH-R1-B, Rhopr-CT/DH-R1-C and Rhopr-CT/DH-R2-B) transiently expressed in HEK293/CNG cell lines**. Dose-dependent effect on the bioluminescence response after addition of Rhopr-CT/DH to HEK293/CNG cells expressing Rhopr-CT/DH-R1-B and Rhopr-CT/DH-R1-C (A) and Rhopr-CT/DH-R2 (B). Vertical bars represent SEM (n=3). Rhopr-CT/DH-R2-B is much more sensitive to Rhopr-CT/DH (EC_50_ = 15nM) compared to Rhopr-CT/DH-R1-B or C (EC_50_ = 200-300nM) and results in a greater response.

To confirm that Rhopr-CT/DH is the ligand for the isolated putative Rhopr-CT/DH-R1 and Rhopr-CT/DH-R2, we expressed these receptors in HEK293/CNG and monitored ligand-receptor interaction using a calcium mobilization assay. Only the receptor isoforms which contained all the 7 transmembrane domains were used in this assay. Rhopr-CT/DH-R1-B and Rhopr-CT/DH-R1-C, which only differ by 2 amino acids in the C-terminus, were both activated by Rhopr-CT/DH with EC_50_ values ranging from 150-300nM (Figure 3A). The maximum luminescence response obtained for both these receptors following the addition of Rhopr-CT/DH was at least 42-fold higher compared to the addition of medium alone. Interestingly, Rhopr-CT/DH-R2-B is much more sensitive to Rhopr-CT/DH (EC_50_ = 15nM) compared to Rhopr-CT/DH-R1-B/C and results in a greater response (191-fold higher than basal response) in our heterologous assay (Figure 3B). None of these receptors were activated by Rhopr-CRF or Drome-PDF (data not shown). Moreover, no response was observed following the addition of Rhopr-CT/DH to the cells that were transfected with empty vector. 

Transfection of Rhopr-CT/DH-R1-A in CHO/G16 cells stably-expressing either Rhopr-CT/DH-R1-B or Rhopr-CT/DH-R1-C did not influence their sensitivity or kinetics of the response following the addition of Rhopr-CT/DH (data not shown). Since we had stably expressed Rhopr-CT/DH-R1-B in CHO/G16 cells we also compared the kinetics of the response in these cells with that of the HEK293/CNG cells. Rhopr-CT/DH produced a rapid response, with the peak response for HEK/CNG cells and CHO/G16 cells between 5-10 seconds and 0-5 seconds, respectively (Figure S3 in File S1).

### Sequence and phylogenetic analysis

**Figure 4:**
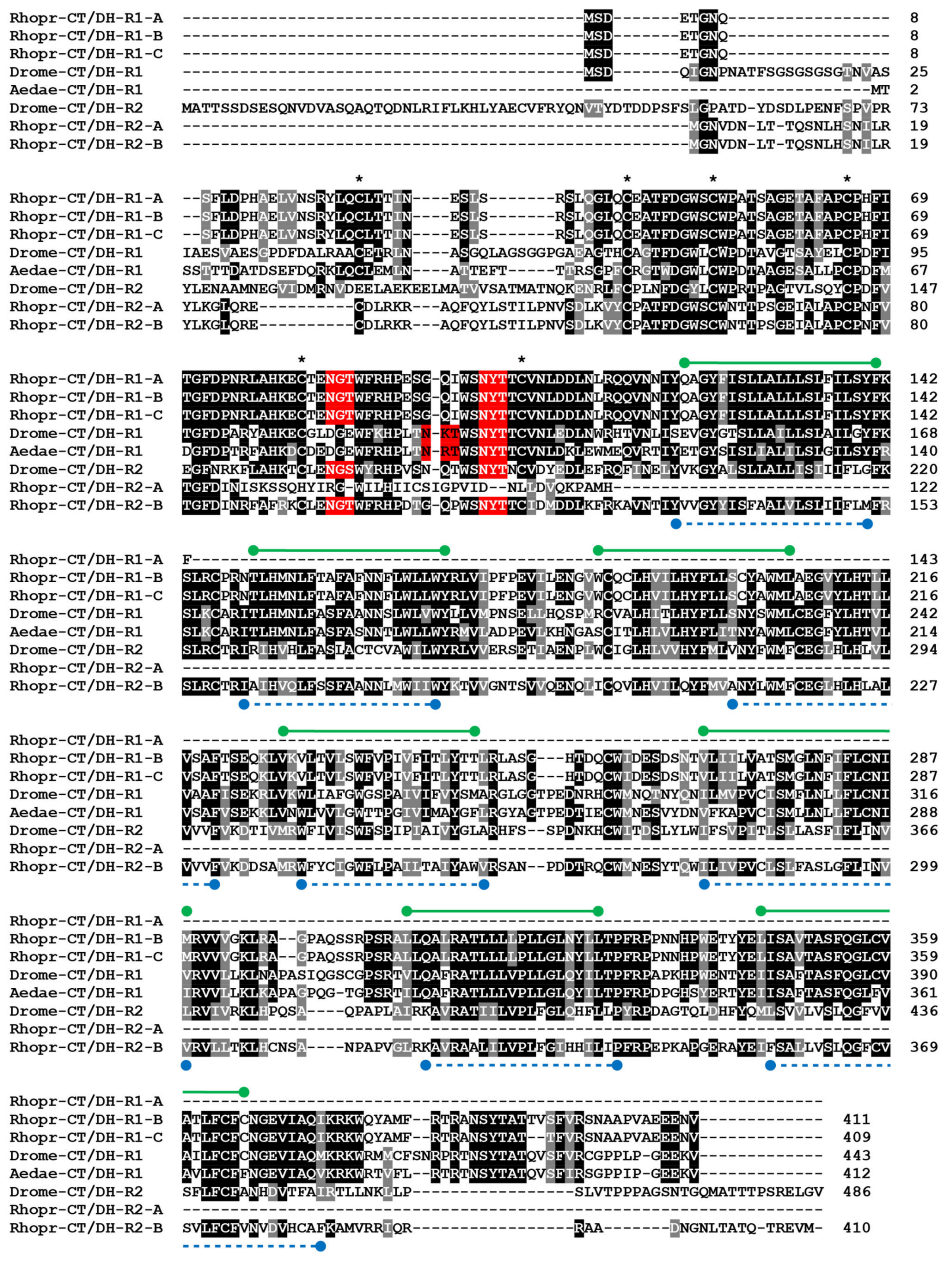
Multiple sequence alignment of select insect **CT**/**DH** receptors. Identical and similar amino acids across 50% of the sequences have been highlighted in black and gray, respectively. The six highly-conserved cysteine residues in N-terminal domain have been marked with an asterisk. Two N-linked glycosylation sites which are conserved across all sequences have been highlighted in red. The predicted locations of the seven transmembrane domains of Rhopr-CT/DH-R1 and Rhopr-CT/DH-R2 have been indicated using green lines and blue dashed lines, respectively.

Rhopr-CT/DH-R amino acid sequences were aligned along with those of Drome-CT/DH-R1, Aedae-CT/DH-R1 (previously referred to as *Aedae*GPCRCAL1) and Drome-CT/DH-R2 (previously an orphan and also referred to as *hector*). The multiple sequence alignment illustrates high conservation across both the receptors (Figure 4). This conservation is localized not only over the seven transmembrane domains but also over the N´-terminal extracellular domain. Positions of the two predicted N-linked glycosylation sites (positions 85 and 100 in Rhopr-CT/DH-R1-A) and the 6 cysteine residues are conserved across most sequences. Since the N´-terminus forms part of the ligand-binding domain, it is not surprising that CT/DH activates both the receptors [28,29]. 

**Figure 5:**
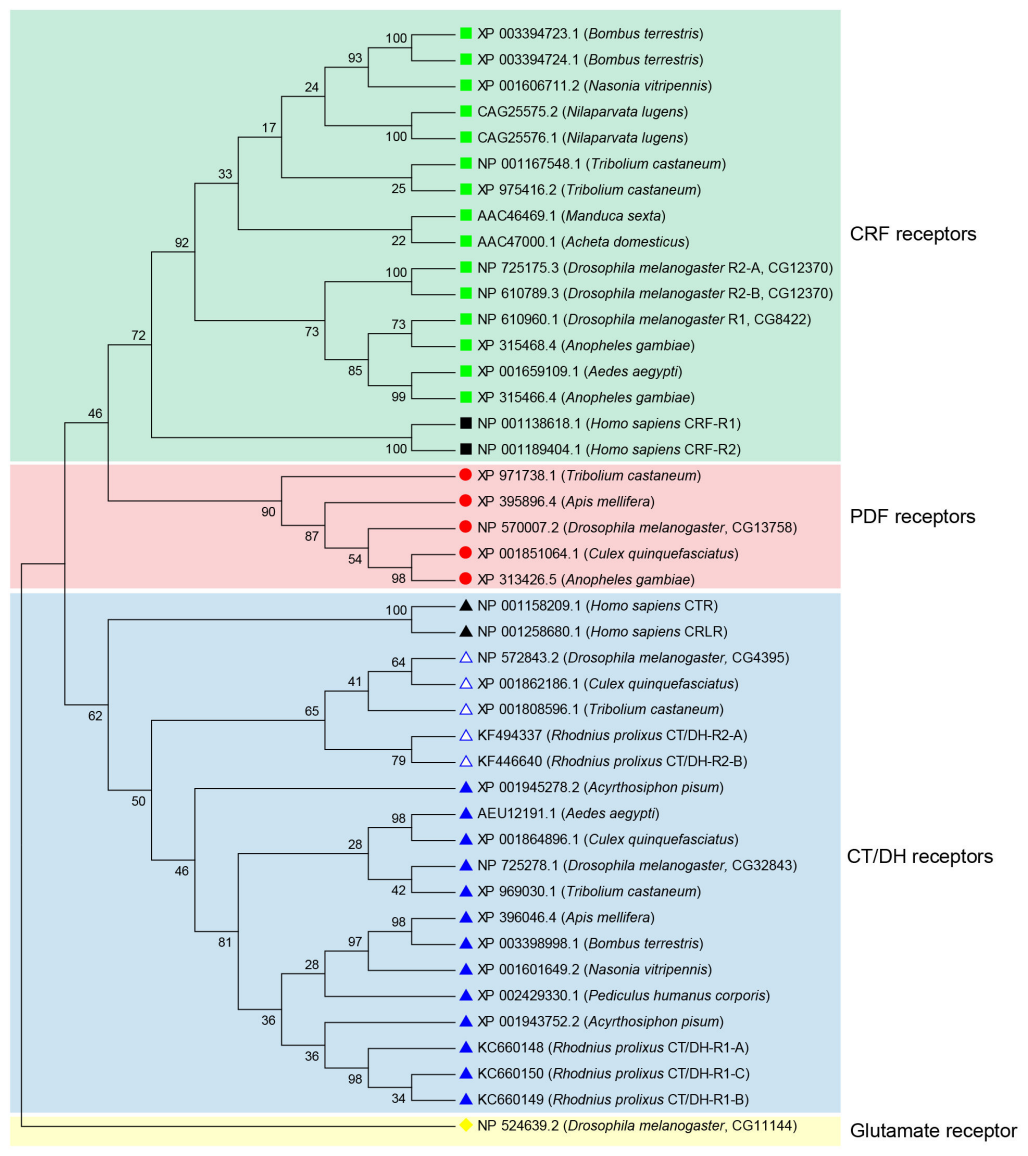
A cladogram of family B1 GPCRs obtained following a maximum parsimonious analysis (1000 bootstrap replicates). The taxa are labelled using GenBank accession numbers and the species names. *Drosophila* *melanogaster* metabotropic glutamate receptor is utilized as an outgroup. Black symbols are used to denote sequences from *Homo* *sapiens* and colored symbols denote insect sequences. Note that the three receptor subtypes (CRF, PDF and CT/DH) form monophyletic clades.

Phylogenetic analysis of family B1 GPCRs reveals three main monophyletic groups (Figure 5). These groups represent the three main receptor types – CRF/DH-Rs, PDF-Rs and CT/DH-Rs. All insect CT/DH-Rs are sister to human CTR and CRLR. This further supports the suggestion that these hormonal systems are evolutionary related. Within the clade comprising CT/DH-Rs, CT/DH-R1s form a monophyletic group and CT/DH-R2s form another monophyletic group, which suggests that these two receptor subtypes arose from a recent duplication in insects, independent from the one in deuterostomes. 

### Spatial expression profile of Rhopr-CT/DH-R transcript variants

**Figure 6:**
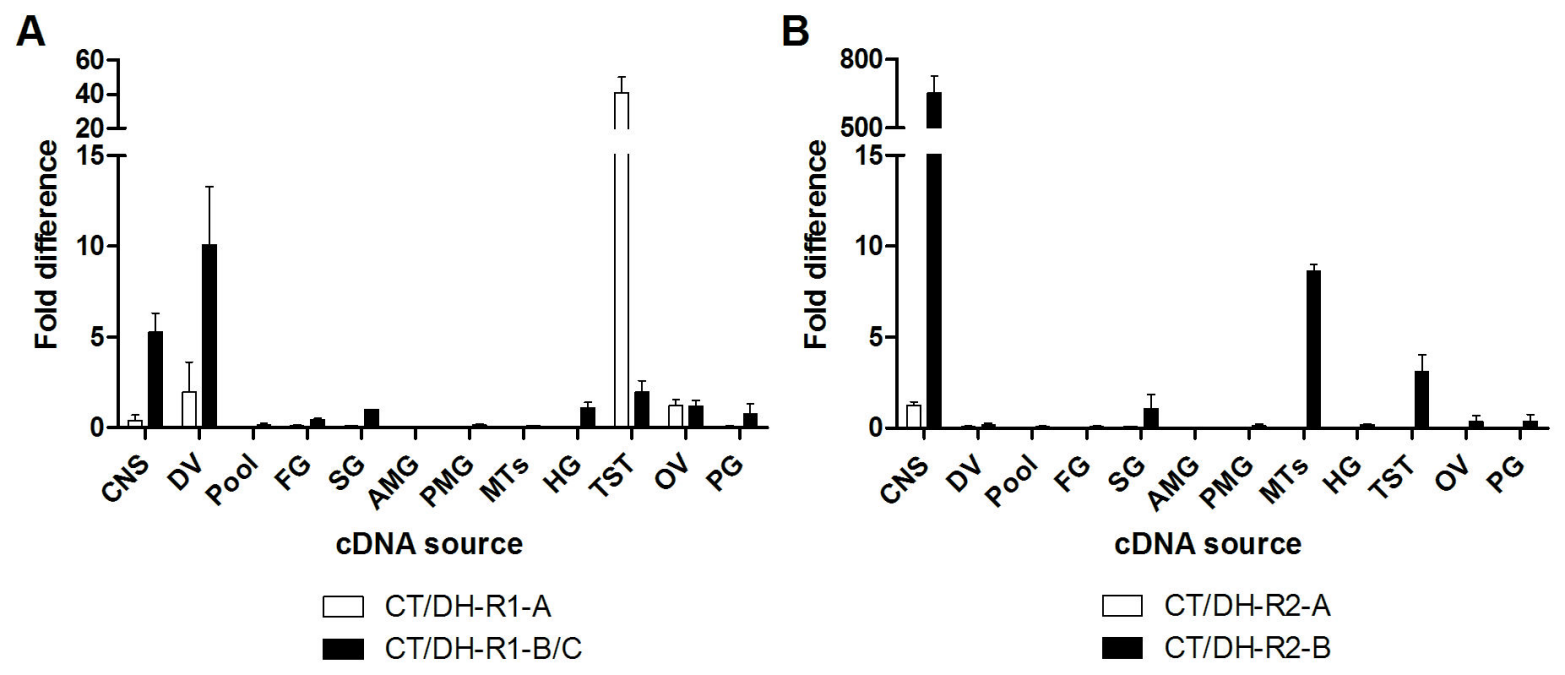
Spatial expression analysis of *Rhopr-CT/DH-Rs* in fifth instar *R*. ***prolixus* determined using quantitative PCR**. *Rhopr-CT/DH-R1* (A) and *Rhopr-CT/DH-R2* (B) expression profile. Expression was analyzed in the following tissues: CNS (central nervous system), DV (dorsal vessel), Pool (fat bodies, abdominal nerves, diaphragms and trachea), FG (foregut), SG (salivary glands), AMG (anterior midgut), PMG (posterior midgut), MTs (Malpighian tubules), HG (hindgut), TST (testes), OV (ovaries) and PG (prothoracic glands). Expression of each variant for both the receptors is shown relative to *Rhopr-CT/DH-R1-B* transcript levels in salivary glands cDNA.

**Figure 7:**
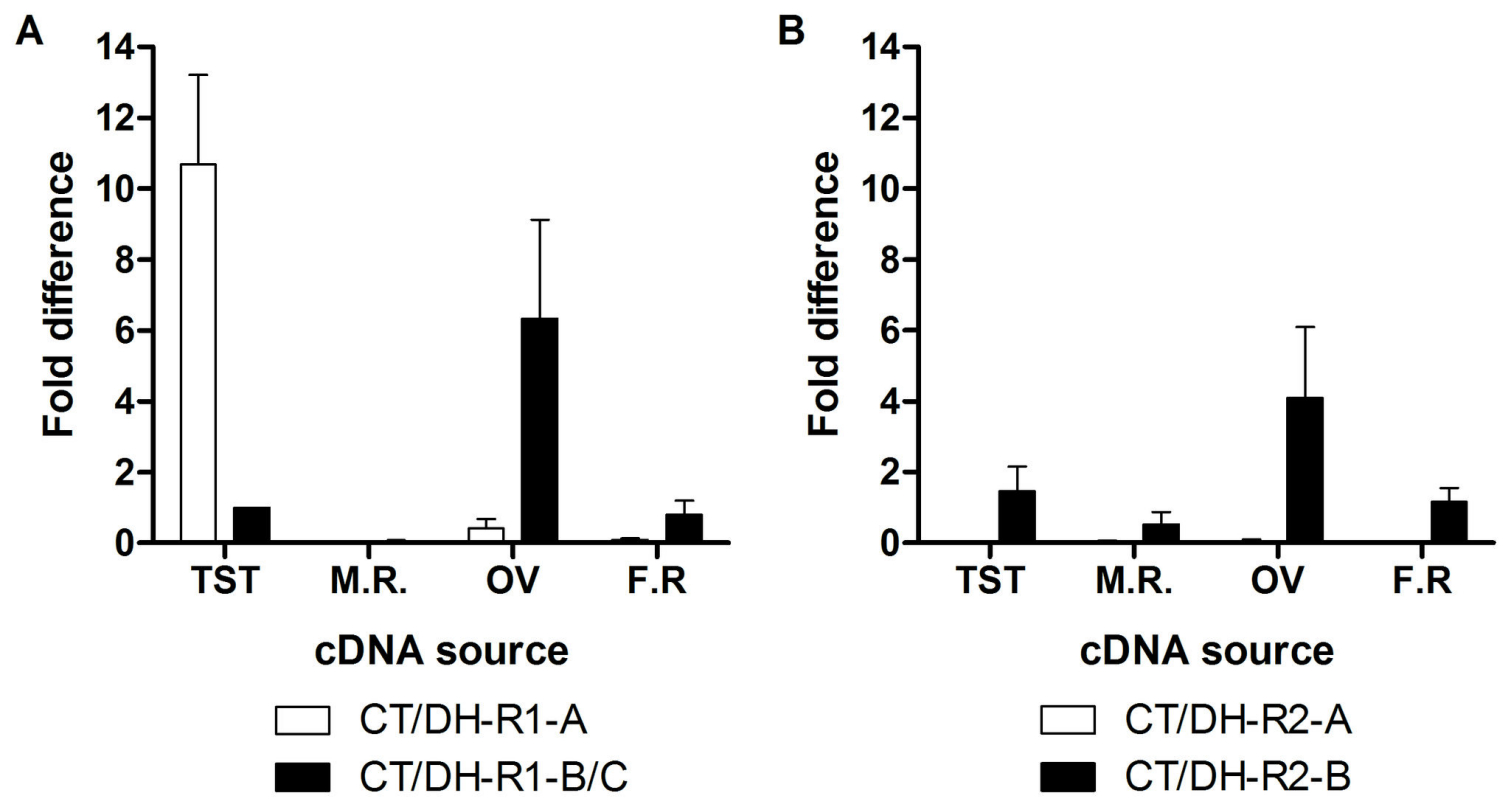
Spatial expression analysis of *Rhopr-CT/DH-Rs* in *R*. ***prolixus* adult reproductive tissues determined using quantitative PCR**. *Rhopr-CT/DH-R1* (A) and *Rhopr-CT/DH-R2* (B) expression profile. Expression was analyzed in the following tissues: TST (testes), M.R. (rest of the male reproductive tissues), OV (ovaries) and F.R. (rest of the female reproductive tissues). Expression of each variant for both the receptors is shown relative to *Rhopr-CT/DH-R1-B* transcript levels in testes cDNA.

In order to discover physiological targets of Rhopr-CT/DH, qPCR analysis was performed to determine the spatial expression pattern of *Rhopr-CT/DH-R* transcript variants (Figure 6). *Rhopr-CT/DH-R1-A*, despite lacking the characteristic seven transmembrane domains, is highly expressed in the testes (Figure 6A). *Rhopr-CT/DH-R1-B/C*, on the other hand, is highly enriched in the CNS and dorsal vessel and expressed at lower levels in the foregut, salivary glands, hindgut, testes, ovaries and prothoracic glands. *Rhopr-CT/DH-R2-A* is only expressed in the CNS and in low amounts (Figure 6B). *Rhopr-CT/DH-R2-B* has the highest abundance in the CNS and this is over 600 fold higher compared to any other Rhopr-CT/DH-R transcript levels in any tissue. Interestingly, *Rhopr-CT/DH-R2-B* and not *Rhopr-CT/DH-R1-B/C*, is expressed in MTs. *Rhopr-CT/DH-R2-B* is also expressed at much lower levels in the salivary glands, testes, ovaries and prothoracic glands. With regards to the adult reproductive tissues, both *Rhopr-CT/DH-R1-B/C* and *Rhopr-CT/DH-R2-B* have the highest expression in ovaries and are expressed at lower levels in the testes and female reproductive tissues minus the ovaries (Figure 7). Similar to the fifth-instar, *Rhopr-CT/DH-R1-A* is highly expressed in the adult testes (Figure 7A). 

## Discussion

In the present study, we have found that *R. prolixus*, and perhaps other insects, contain two CT/DH-Rs. *Rhopr-CT/DH-R1* encodes three splice variants while *Rhopr-CT/DH-R2* encodes two splice variants. We functionally characterized full-length receptor isoforms encoded by these transcript variants in HEK293/CNG cells. The assay used to characterize these receptors monitored calcium mobilization into the cells from the extracellular media. Since Rhopr-CT/DH is thought to mediate its effects via cAMP as the secondary messenger, its binding to the receptor would result in receptor activation and a subsequent increase in intracellular cAMP levels [7]. This cAMP would then bind to the CNG channel, resulting in its opening and an influx of calcium from the extracellular medium. The amount of calcium mobilized was detected using the reporter, aequorin. Using this assay, we confirmed that Rhopr-CT/DH-R1-B, Rhopr-CT/DH-R1-C and Rhopr-CT/DH-R2-B were all activated by Rhopr-CT/DH. This work therefore essentially de-orphans the *D. melanogaster hector* and related receptors in other insects. Responses that were robust (ranging from 42 to 191-fold higher than basal response) and sensitive (EC_50_ values in the low nanomolar range) were obtained in this cell line. This is surprising because according to a previous report, HEK293 cells expressing Drome-CT/DH-R1 were not responsive to Drome-CT/DH until RCP, an accessory protein, was co-expressed with the receptor [12]. Even after the expression of *D. melanogaster* RCP along with the receptor, the maximum response obtained was under 2-fold that of the basal response. An improved sensitivity (EC_50_ = 82nM) and a greater response (7-fold) to Drome-CT/DH was only observed when the human RCP and RAMPs were expressed along with the receptor. This led to the conclusion that perhaps RCP and RAMPs are required for signalling through insect CT/DH-Rs just like mammalian calcitonin receptors; however, our results with a similar cell line, coupled with the fact that proteins with sequence homology to human RCP and RAMPs are not found in the *R. prolixus* genome, suggests that Rhopr-CT/DH-Rs may not require accessory proteins for effective signalling. 

Comparing the responses of the two Rhopr-CT/DH-Rs, Rhopr-CT/DH-R2-B is at least 10-fold more sensitive to Rhopr-CT/DH than are Rhopr-CT/DH-R1-B or Rhopr-CT/DH-R1-C; the EC_50_ values of Rhopr-CT/DH-R1-B and Rhopr-CT/DH-R1-C range from 150-300nM. These values are still relatively high and hence we don’t rule out the possibility that other endogenous ligands may also activate this receptor. Another possibility is that Rhopr-CT/DH-R1 and Rhopr-CT/DH-R2 may interact as heterodimers as is so common of family B1 GPCRs [30]. Either way, Rhopr-CT/DH-R1-B and Rhopr-CT/DH-R1-C do not seem to be functionally different. *Rhopr-CT/DH-R1-A* is highly expressed in the testes and such high expression may suggest a role for this truncated receptor isoform rather than it being just an error in splicing. Since *Rhopr-CT/DH-R1-A* and *Rhopr-CT/DH-R1-B/C* are expressed in the CNS, dorsal vessel, testes and ovaries, we questioned whether Rhopr-CT/DH-R1-A could interact with Rhopr-CT/DH-R1-B and Rhopr-CT/DH-R1-C and affect their signalling. If Rhopr-CT/DH-R1-A influenced (either inhibited or stimulated) the surface expression of Rhopr-CT/DH-R1-B/C, one would expect their EC_50_ values to be altered. However, Rhopr-CT/DH-R1-A did not influence the signalling through Rhopr-CT/DH-R1-B and Rhopr-CT/DH-R1-C in our heterologous assay utilizing CHO/G16 cells. Hence, the role of Rhopr-CT/DH-R1-A, if any, is still unclear. 

Spatial expression analysis of the two receptors using qPCR demonstrates that they have distinct expression patterns, with both receptors expressed centrally and peripherally. We utilized *Rhopr-CT/DH-R1-B* transcript levels in salivary glands and testes cDNA to show the relative expression of each variant for both the receptors in fifth-instar tissues and adult reproductive tissues, respectively. We acknowledge that this method is not as effective as absolute quantification to compare the expression of two different genes and hence this comparison between the two receptors is only an approximate. Nonetheless, at least one of *Rhopr-CT/DH-R1-B*, *Rhopr-CT/DH-R1-C* or *Rhopr-CT/DH-R2-B* is expressed in the dorsal vessel, salivary glands, hindgut and MTs which have previously been shown to be targets of Rhopr-CT/DH [5,7,9]. Rhopr-CT/DH causes, at most, a 17-fold increase in the rate of fluid secretion by MTs compared to saline controls [5]; however, this rate is only 1.5% of the maximum rate stimulated by serotonin and Rhopr-CRF/DH. Perhaps Rhopr-CT/DH is used by the insect at times when diuresis needs to be maintained at a low level, such as the period after the rapid post-feeding diuresis and during digestion. Based on the expression patterns, Rhopr-CT/DH-R2 appears to be the one responsible for MT secretion in *R. prolixus*, whereas CT/DH-R1 is responsible for the diuretic function in *D. melanogaster* and *A. aegypti* [12,15]. FlyAtlas (www.flyatlas.org) data indicates that *Drome-CT/DH-R2* is not expressed in *D. melanogaster* MTs. This observation suggests that the specialized functions, diuresis mediated through CT/DH-R2 in *R.prolixus* and through CT/DH-R1 in dipterans, have evolved independently. The phylogeny of these two receptor clusters has been discussed previously [31]. It remains to be seen whether CT/DH-R2 mediates diuresis in other non-dipteran species. Rhopr-CT/DH also increases muscle contractions of dorsal vessel, salivary glands and hindgut, all of which indicate a role for Rhopr-CT/DH in feeding-related physiological events [7,9]. For instance, the contraction of salivary glands would aid in the release of saliva at the time of feeding. An increase in dorsal vessel contractions would result in increased circulation of the haemolymph, as well as the diuretic hormones that are present in the haemolymph following feeding [32]. Hindgut contractions aid in expulsion of waste, reduce unstirred layers around MTs and also increase haemolymph circulation. Hence increased contractility of dorsal vessel and hindgut could indirectly aid in post-feeding diuresis. 

All the receptor transcripts are expressed with in the CNS but it is unclear which neural circuits Rhopr-CT/DH-Rs may be involved with. The qPCR analysis also identified novel target tissues of Rhopr-CT/DH; these include testes, ovaries and prothoracic glands. Rhopr-CT/DH, thus, may regulate reproductive physiology and ecydsteroidogenesis. CT/DH-like immunoreactivity is not associated with male or female reproductive tissues of *R. prolixus* (Zandawala and Orchard, unpublished). Hence the effect, if any, of Rhopr-CT/DH on reproductive tissues will most-likely be mediated via a hormonal route. *Drome-CT/DH-R2* (*hector*) is expressed in a subset of *fruitless* neurons and has been shown to be critical for male courtship [33]. Consistent with this role, its transcript is enriched in *D. melanogaster* brain and male accessory glands [34]. It remains to be examined if Rhopr-CT/DH-R2 is also involved in courtship behaviour considering its expression in the CNS and reproductive tissues. 

This is the first study to reveal that insects possess at least two CT/DH receptors, which may be functionally different. Our expression analysis suggests that Rhopr-CT/DH-Rs may mediate feeding-related physiological events, some of which must await further investigation. Moreover, we also identified novel target tissues for this neuropeptide, including testes, ovaries and prothoracic glands, suggesting a possible role for Rhopr-CT/DH in reproductive physiology and development. 

## Supporting Information

File S1 **Tables S1-S6 and Figures S1-S3.**Table S1: Primers used to amplify the partial cDNA sequence for *Rhopr-CT/DH-R1* and *Rhopr-CT/DH-R2*. Table S2: Primers used to perform 5’ RACE PCR reactions. Table S3: Primers used to perform 3’ RACE PCR reactions. Table S4: Primers used to amplify the largest cDNA fragments. Table S5: Primers used to amplify full ORF and introduce Kozak sequence. Table S6: Primers used for qPCR reactions. Figure S1: *Rhopr-CT/DH-R1-A* cDNA sequence and the deduced amino acid sequence. The numbering for each sequence is shown at right. Within the nucleotide sequence, the exon-exon boundaries are shaded in gray and the potential polyadenylation signal is double-underlined. Within the amino acid sequence, the initial methionine start codon has been capitalized, the six conserved cysteine residues are shaded in red, the potential N-linked glycosylation sites are boxed and the predicted transmembrane domain is underlined. Figure S2: *Rhopr-CT/DH-R2-A* cDNA sequence and the deduced amino acid sequence. The numbering for each sequence is shown at right. Within the nucleotide sequence, the exon-exon boundaries are shaded in gray. Within the amino acid sequence, the initial methionine start codon has been capitalized, the conserved cysteine residues are shaded in red and the potential N-linked glycosylation sites are boxed. Figure S3: Kinetics of the bioluminescence responses of HEK/CNG (A) and CHO/G16 (B) cells expressing Rhopr-CT/DH-R1-B. Bioluminescence was recorded for every 5 seconds for 15 seconds following the addition of phosphate-buffered saline (PBS) or 10^-6^M peptide. Vertical bars represent SEM (n=3). Rhopr-CT/DH produced a rapid response, with the peak response for HEK/CNG cells and CHO/G16 cells between 5-10 seconds and 0-5 seconds, respectively. The assay was performed using the methods described earlier.
(DOCX)
