# Supplementary material for: Isolation and Functional Characterization of Calcitonin-Like Diuretic Hormone Receptors in Rhodnius prolixus
Source: PLoS One. 2013 Nov 29;8(11):e82466. doi: 10.1371/journal.pone.0082466 (PMC3843727; doi:10.1371/journal.pone.0082466)
Supplement: File S1 — Tables S1-S6 and Figures S1-S3.Table S1: Primers used to amplify the partial cDNA sequence for Rhopr-CT/DH-R1 and Rhopr-CT/DH-R2. Table S2: Primers used to perform 5’ RACE PCR reactions. Table S3: Primers used to perform 3’ RACE PCR reactions. Table S4: Primers used to amplify the largest cDNA fragments. Table S5: Primers used to amplify full ORF and introduce Kozak sequence. Table S6: Primers used for qPCR reactions. Figure S1: Rhopr-CT/DH-R1-A cDNA sequence and the deduced amino acid sequence. The numbering for each sequence is shown at right. Within the nucleotide sequence, the exon-exon boundaries are shaded in gray and the potential polyadenylation signal is double-underlined. Within the amino acid sequence, the initial methionine start codon has been capitalized, the six conserved cysteine residues are shaded in red, the potential N-linked glycosylation sites are boxed and the predicted transmembrane domain is underlined. Figure S2: Rhopr-CT/DH-R2-A cDNA sequence and the deduced amino acid sequence. The numbering for each sequence is shown at right. Within the nucleotide sequence, the exon-exon boundaries are shaded in gray. Within the amino acid sequence, the initial methionine start codon has been capitalized, the conserved cysteine residues are shaded in red and the potential N-linked glycosylation sites are boxed. Figure S3: Kinetics of the bioluminescence responses of HEK/CNG (A) and CHO/G16 (B) cells expressing Rhopr-CT/DH-R1-B. Bioluminescence was recorded for every 5 seconds for 15 seconds following the addition of phosphate-buffered saline (PBS) or 10-6M peptide. Vertical bars represent SEM (n=3). Rhopr-CT/DH produced a rapid response, with the peak response for HEK/CNG cells and CHO/G16 cells between 5-10 seconds and 0-5 seconds, respectively. The assay was performed using the methods described earlier. (DOCX) [file pone.0082466.s001.docx]

**Supplementary information**

**Table S1**: Primers used to amplify the partial cDNA sequence for *Rhopr-CT/DH-R1* and *Rhopr-CT/DH-R2.*

| Primer | Tm | Sequence (5’-3’) |
| --- | --- | --- |
| *Rhopr-CT/DH-R1* | | |
| DH31R1FOR1 | 62.1 | TCTGTGGCTTCTCTGGTATAGG |
| DH31R1REV1 | 62.1 | AAGTTACTTGGAAGGACGCTG |
| *Rhopr-CT/DH-R2* | | |
| DH31R2FOR1 | 62.9 | GTTTGCCGCCAATAATCTG |
| DH31R2REV1 | 62.8 | CCTGTAAAGAAACAAGAAGTGCTG |

**Table S2**: Primers used to perform 5’ RACE PCR reactions.

| Primer | Tm | Sequence (5’-3’) |
| --- | --- | --- |
| Plasmid-specific primers | | |
| DNR-LIB FOR1 | 63.9 | GTGGATAACCGTATTACCGCC |
| DNR-LIB FOR2 | 64.5 | ACGGTACCGGACATATGCC |
| *Rhopr-CT/DH-R1* | | |
| DH31R1-5RACE-REV1 | 64.0 | CATACACCGTTTTCCAGAATTACC |
| DH31R1-5RACE-REV2 | 64.2 | CAGAATTACCTCCGGAAATGG |
| DH31R1-5RACE-REV3 | 64.4 | ACCAGCCTATACCAGAGAAGCC |
| DH31R1-5RACE-REV4 | 63.8 | ACCAAATTTGTCCAGATTCAGG |
| DH31R1-5RACE-REV5 | 64.3 | GATTCAGGATGTCTAAACCATGTACC |
| DH31R1-5RACE-REV6 | 64.5 | GTTGTTAGACATTGAAGGTAGCGTG |
| DH31R1-5RACE-REV7 | 65.2 | AGTTCAGCATGTGGGTCCAG |
| *Rhopr-CT/DH-R2* | | |
| DH31R2-5RACE-REV1 | 64.4 | CAAGATGTAGATGCAATCCTTCG |
| DH31R2-5RACE-REV2 | 64.5 | CAATCCTTCGCAGAACATCC |
| DH31R2-5RACE-REV3 | 64.7 | ATCAGATTATTGGCGGCAAAC |
| DH31R2-5RACE-REV4 | 63.8 | ATGTCGCTGGGCAGTACAC |
| DH31R2-5RACE-REV6 | 64.2 | TGTTGACTGCTTTTCGAAACTTAAG |
| DH31R2-5RACE-REV7 | 64.4 | TGTCAATGCAGGTCGTATAATTTG |
| DH31R2-5RACE-REV8 | 63.2 | GTAGTATTCCAACATGACCAGCC |

**Table S3**: Primers used to perform 3’ RACE PCR reactions.

| Primer | Tm | Sequence (5’-3’) |
| --- | --- | --- |
| Plasmid-specific primers | | |
| pDNR-LIB 3 -25 REV | 63.6 | GCCAAACGAATGGTCTAGAAAG |
| pDNR-LIB 3 -88 REV | 63.3 | AGTCATACCAGGATCTCCTAGGG |
| *Rhopr-CT/DH-R1* | | |
| DH31R1-3RACE-FOR1 | 63.6 | GGTTGTTGGTAAGTTAAGAGCTGG |
| DH31R1-3RACE-FOR2 | 64.2 | GACCTTCAAGAGCCTTACTACAAGC |
| DH31R1-3RACE-FOR3 | 64.9 | GCTTGGGCTTAACTATCTTTTGACTC |
| *Rhopr-CT/DH-R2* | | |
| DH31R2-3RACE-FOR1 | 64.1 | TTGGTCTTAGGAAGGCTGTACG |
| DH31R2-3RACE-FOR2 | 63.9 | GAAAGCACCAGGAGAACGAG |
| DH31R2-3RACE-FOR3 | 64.4 | TCAGCACTTCTTGTTTCTTTACAGG |

**Table S4**: Primers used to amplify the largest cDNA fragments.

| Primer | Tm | Sequence (5’-3’) |
| --- | --- | --- |
| *Rhopr-CT/DH-R1* | | |
| DH31-R1-FOR2 | 62.1 | GAAGTTGTGCAAAGTTTGTGG |
| DH31-R1-REV3 | 61.5 | TGTGAAACATCTAATGGACAAAAC |
| *Rhopr-CT/DH-R2* | | |
| DH31R2FOR5 | 55.5 | ACCACCTCCGAGTACAC |
| DH31R2REV3 | 54.3 | CCTTACAACAACATATAATTCATA |

**Table S5**: Primers used to amplify full ORF and introduce Kozak sequence.

| Primer | Tm | Sequence (5’-3’) |
| --- | --- | --- |
| *Rhopr-CT/DH-R1* | | |
| DH31R1-KOZAK-FOR | 62.4 | GCCACCATGTCGGATG |
| DH31R1-ORF-REV | 60.8 | CTACATTATTTTATATTGAGACCCATTTC |
| DH31-R1-A-REV1 | 60.4 | AAATAACTTCCTGGCCATTG |
| *Rhopr-CT/DH-R2-B* | | |
| DH31R2-KOZAK-FOR | 61.0 | GCCACCATGAGAAATGTAGAC |
| DH31R2-ORF-REV | 60.6 | CGAAATGATCTAGTCTCGCA |

**Table S6**: Primers used for qPCR reactions.

| Primer | Tm | Sequence (5’-3’) |
| --- | --- | --- |
| *Rhopr-CT/DH-R1-A* | | |
| qPCR-DH31R-A-FOR1 | 56.1 | GATGATTTAAATTTAAGGCAAC |
| qPCR-DH31R-A-REV1 | 56.5 | GGACATCTTAAAGATTTTTATTCTAC |
| *Rhopr-CT/DH-R1-B/C* | | |
| qPCR-DH31R-B-FOR2 | 58.1 | CTTTGATCCAAATCGATTG |
| qPCR-DH31R-B-REV1 | 56.0 | CATCTTAAAGATTTAAAATAAGATAGG |
| *Rhopr-CT/DH-R2-A* | | |
| qPCR-DH31R2A-FOR | 61.7 | CAGGATTTGATATAAATATTTCGAAAAG |
| qPCR-DH31R2A-REV | 61.7 | TGGCAAATTAACTGATTTTCTTG |
| *Rhopr-CT/DH-R2-B* | | |
| qPCR-DH31R2B-FOR2 | 61.4 | TCGCAAATGTTTAGAAAATGG |
| qPCR-DH31R2A-REV | 61.7 | TGGCAAATTAACTGATTTTCTTG |
| *Rhopr-alpha-tubulin* | | |
| alphaTUB-qPCR-F | 64.3 | GTGTTTGTTGATTTGGAACCTACAG |
| alphaTUB-qPCR-R | 64.4 | CCGTAATCAACAGACAATCTTTCC |
| *Rhopr-beta-actin­* | | |
| Actin5c-qPCR-F | 62.9 | AGAGAAAAGATGACGCAGATAATGT |
| Actin5c-qPCR-R | 63.6 | ATATCCCTAACAATTTCACGTTCG |
| *Rhopr-ribosomal protein 49* | | |
| rp49-qPCR-F | 63.7 | GTGAAACTCAGGAGAAATTGGC |
| rp49-qPCR-R | 65.0 | AGGACACACCATGCGCTATC |


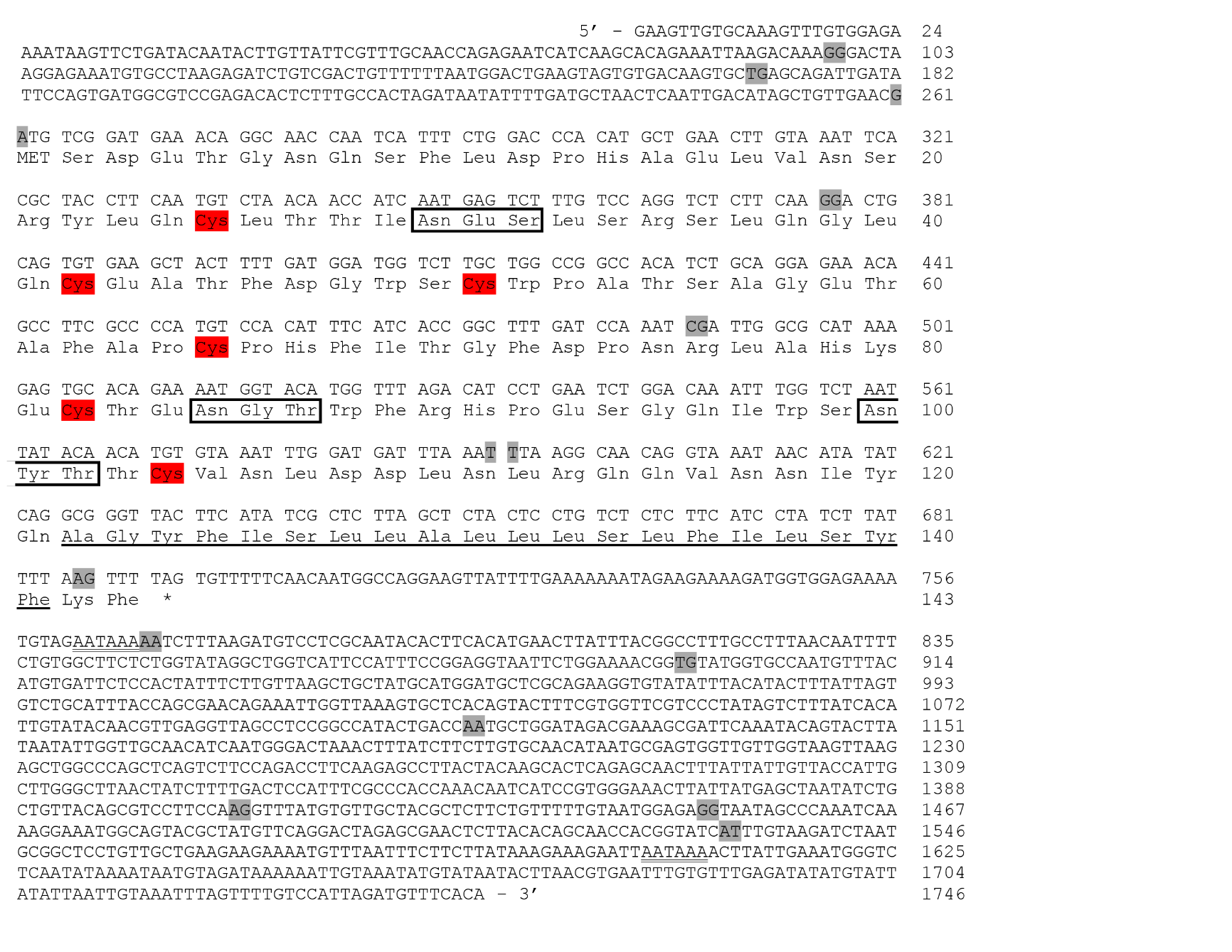


**Figure S1**: *Rhopr-CT/DH-R1-A* cDNA sequence and the deduced amino acid sequence. The numbering for each sequence is shown at right. Within the nucleotide sequence, the exon-exon boundaries are shaded in gray and the potential polyadenylation signal is double-underlined. Within the amino acid sequence, the initial methionine start codon has been capitalized, the six conserved cysteine residues are shaded in red, the potential N-linked glycosylation sites are boxed and the predicted transmembrane domain is underlined.


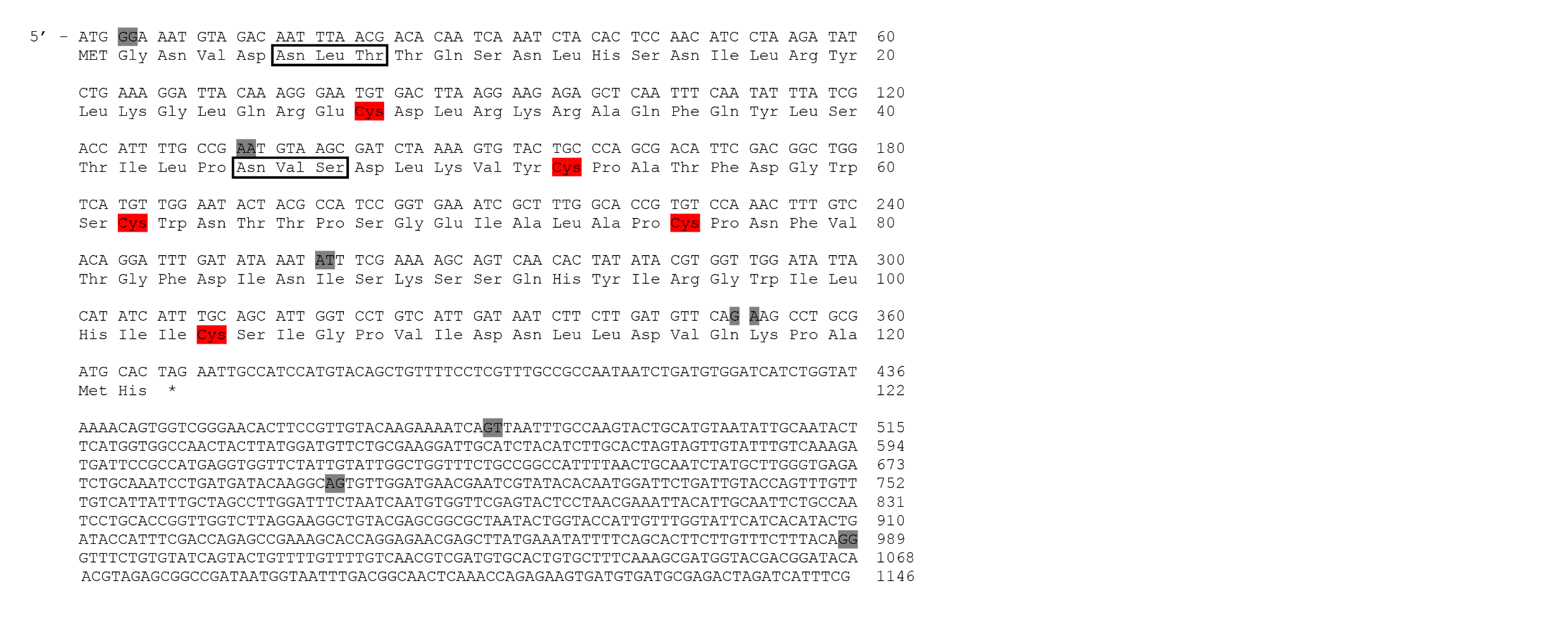


**Figure S2**: *Rhopr-CT/DH-R2-A* cDNA sequence and the deduced amino acid sequence. The numbering for each sequence is shown at right. Within the nucleotide sequence, the exon-exon boundaries are shaded in gray. Within the amino acid sequence, the initial methionine start codon has been capitalized, the conserved cysteine residues are shaded in red and the potential N-linked glycosylation sites are boxed.


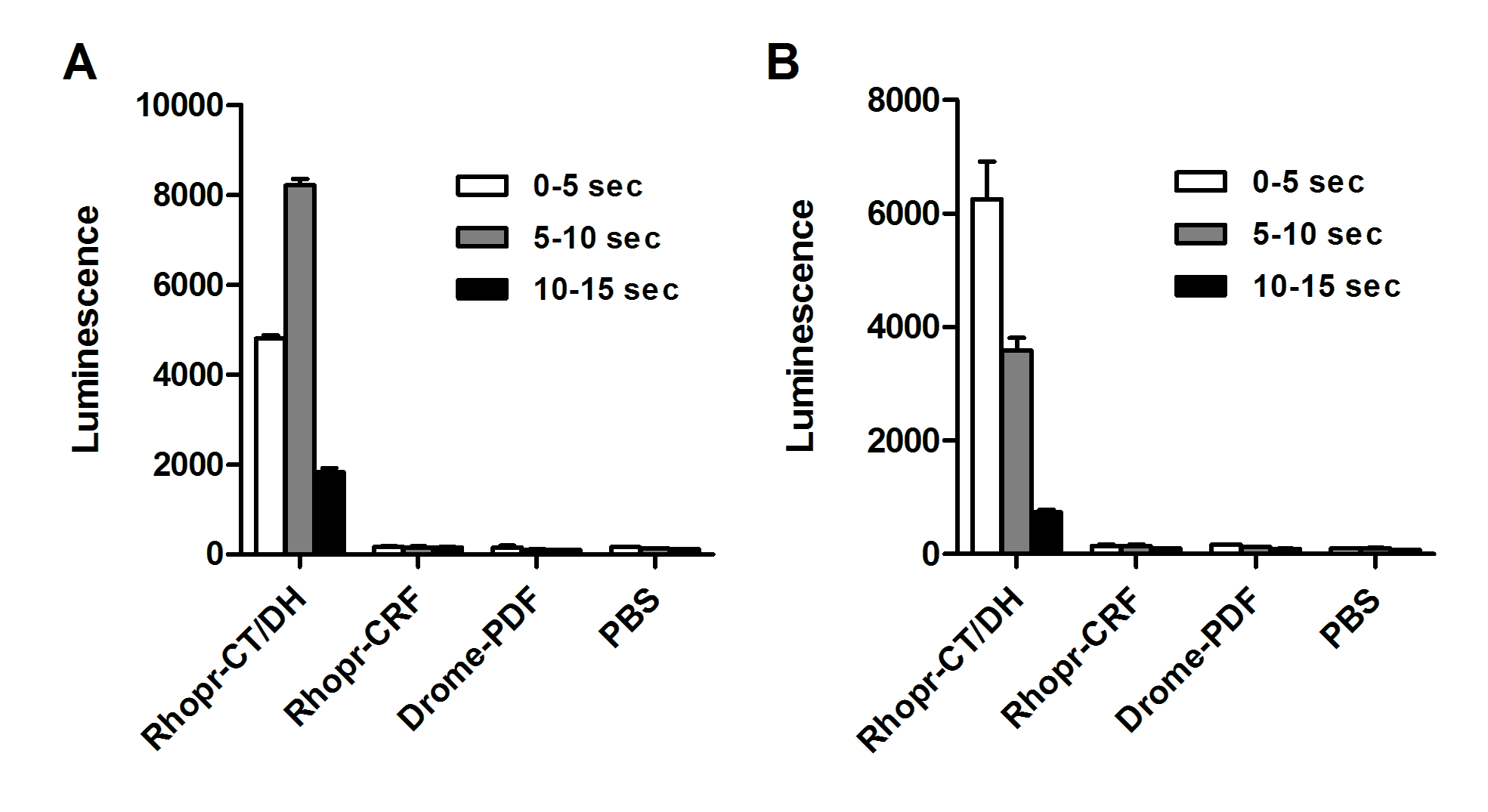


**Figure S3:** Kinetics of the bioluminescence responses of HEK/CNG (A) and CHO/G16 (B) cells expressing Rhopr-CT/DH-R1-B. Bioluminescence was recorded for every 5 seconds for 15 seconds following the addition of phosphate-buffered saline (PBS) or 10^-6^M peptide. Vertical bars represent SEM (n=3). Rhopr-CT/DH produced a rapid response, with the peak response for HEK/CNG cells and CHO/G16 cells between 5-10 seconds and 0-5 seconds, respectively. The assay was performed using the methods described earlier [[1](#_ENREF_1),[2](#_ENREF_2)].

1. Stables J, Green A, Marshall F, Fraser N, Knight E, et al. (1997) A bioluminescent assay for agonist activity at potentially any G-protein-coupled receptor. Anal Biochem 252: 115-126.

2. Staubli F, Jorgensen TJ, Cazzamali G, Williamson M, Lenz C, et al. (2002) Molecular identification of the insect adipokinetic hormone receptors. Proc Natl Acad Sci U S A 99: 3446-3451.
